# Decreased levels of baseline and drug-induced tubulin polymerisation are hallmarks of resistance to taxanes in ovarian cancer cells and are associated with epithelial-to-mesenchymal transition

**DOI:** 10.1038/bjc.2017.102

**Published:** 2017-04-11

**Authors:** George E Duran, Yan C Wang, François Moisan, E Brian Francisco, Branimir I Sikic

**Affiliations:** 1 Department of Medicine (Oncology), Stanford University School of Medicine, Stanford, CA 94305, USA

**Keywords:** non-MDR, drug resistance, taxanes, EMT, ovarian cancer, tubulin polymerisation

## Abstract

**Background:**

*ABCB1* expression is uncommon in ovarian cancers in the clinical setting so we investigated non-MDR mechanisms of resistance to taxanes.

**Methods:**

We established eight taxane-resistant variants from the human ovarian carcinoma cell lines A2780/1A9, ES-2, MES-OV and OVCAR-3 by selection with paclitaxel or docetaxel, with counter-selection by the transport inhibitor valspodar.

**Results:**

Non-MDR taxane resistance was associated with reduced intracellular taxane content compared to parental controls, and cross-resistance to other microtubule stabilising drugs. Collateral sensitivity to depolymerising agents (vinca alkaloids and colchicine) was observed with increased intracellular vinblastine. These variants exhibited marked decreases in basal tubulin polymer and in tubulin polymerisation in response to taxane exposure. TUBB3 content was increased in 6 of the 8 variants. We profiled gene expression of the parental lines and resistant variants, and identified a transcriptomic signature with two highly significant networks built around *FN1* and *CDKN1A* that are associated with cell adhesion, cell-to-cell signalling, and cell cycle regulation. miR-200 family members miR-200b and miR-200c were downregulated in resistant cells, associated with epithelial to mesenchymal transition (EMT), with increased *VIM*, *FN1*, *MMP2* and/or *MMP9.*

**Conclusions:**

These alterations may serve as biomarkers for predicting taxane effectiveness in ovarian cancer and should be considered as therapeutic targets.

The taxane drugs docetaxel and paclitaxel are important chemotherapeutic agents for ovarian cancers, and act by stabilising microtubules, leading to cell cycle arrest or apoptosis via p53-dependent or -independent pathways (Horwitz, 1994; Dumontet and Sikic, 1999; Xiao *et al*, 2006).

The human *ABCB1* gene, which encodes the multidrug resistance (MDR) transporter P-glycoprotein (P-gp), is expressed in the minority of ovarian cancers at diagnosis, but has been shown to be an adverse prognostic factor in some though not all studies (Baekelandt *et al*, 2000; Materna *et al*, 2004; Penson *et al*, 2004; Hedditch *et al*, 2014; Sedlakova *et al*, 2015). Although P-gp confers a high level of cellular resistance to many anticancer drugs, many other drug resistance mechanisms are thought to play a role in determining cellular response to taxanes including alterations of microtubule structure and dynamics, tubulin isotypes, and factors that regulate programmed cell death (Dumontet *et al*, 1996; Dumontet and Sikic, 1999; Goncalves *et al*, 2001; Orr *et al*, 2003; Esteve *et al*, 2006; Tommasi *et al*, 2007).

We have established two sets of taxane-resistant variants, one set selected in taxanes alone which express *ABCB1*/P-gp (Wang *et al*, 2006), and another set of non-MDR variants exposed to taxanes in the presence of the P-gp inhibitor valspodar. Microarray analysis was applied to all taxane-resistant variants to explore new genetic markers and mechanisms that might be useful in the prediction of sensitivity and resistance to taxane therapy. In this report, we also analysed the tubulin isotype composition and microtubule polymerisation before and after taxane drug exposure in the parental cell lines and non-MDR drug-resistant variants.

## Materials and methods

### Drugs and chemicals

Docetaxel was a gift from Sanofi Oncology (Bridgewater, NJ, USA), Novartis Pharmaceuticals provided the P-gp inhibitor valspodar (PSC-833, PSC; East Hanover, NJ, USA), and the other anticancer drugs used in this study were obtained from the drug repository of the National Cancer Institute (Bethesda, MD, USA). Drug stock solutions were prepared in absolute ethanol at 1 mmol l^−1^. Other chemicals were purchased from Sigma-Aldrich (St Louis, MO, USA).

### Cell lines and the development of taxane-resistance variants

We analysed 18 ovarian carcinoma cell lines for taxane sensitivity, *ABCB1* expression, and growth characteristics. Four lines (3 serous: A2780/1A9, MES-OV and OVCAR-3; and one clear cell: ES-2) were chosen as parental lines for the generation of taxane-resistant variants, and cultured as described previously (Wang *et al*, 2006). These lines were chosen because they were the most sensitive to taxanes, did not express high levels of *ABCB1*, and grew readily *in vitro* (doubling times of ⩽48 h in standard tissue culture media). The 3 parental serous lines did not express P-gp, and the clear cell line expressed very low but detectable levels.

Each cell line was exposed to docetaxel or paclitaxel at IC_50_ levels (the concentration required to kill 50% of the population), with or without the P-gp inhibitor valspodar (2 *μ*mol l^−1^). The concentration of the taxanes used for selection of drug resistance was gradually increased until the resulting variants displayed at least a 10-fold resistance to the selecting agents. The results of selection by taxanes without a P-gp inhibitor, resulting in resistant variants expressing high levels of P-gp, have been published (Wang *et al*, 2006).

### SRB cytotoxicity assay

Cytotoxicity of drugs was determined using a 72 h sulforhodamine B (SRB) assay (Spicakova *et al*, 2010). Each drug was tested in quadruplicate, with each experiment reproduced at least three times. The IC_50_ values were determined directly from semi-logarithmic dose–response curves generated using the Hill equation in Kaleidagraph software (Synergy Software, Reading, PA, USA).

### Tubulin polymerisation assay

Soluble and polymerised tubulin fractions were isolated as previously reported (Duran *et al*, 2015), and the percentage of tubulin polymer present in each fraction was calculated on the basis of total tubulin (soluble and polymer) present in each experimental condition as determined by densitometry.

### Flow cytometric assessment of intracellular BODIPY-labeled drugs

The accumulation and drug content of fluorescent BODIPY-paclitaxel and BODIPY-vinblastine (both Thermo Fisher Scientific, Carlsbad, CA, USA) was determined by flow cytometry. Cells were harvested, exposed to drug for 1 h at 37 °C, drug was removed by centrifugation (200 *g*), and washed twice in cold PBS. For intracellular drug content, cells were allowed to efflux for an additional hour after accumulation, and were washed several times to ensure that no unbound intracellular drug remained. Drug was detected using an LSR II flow cytometer (BD Biosciences, San Jose, CA, USA).

### Microarray preparation

mRNA from ovarian cancer cell lines was isolated using the FastTrack kit (Invitrogen Life Technologies, Carlsbad, CA, USA) and hybridised to Stanford human cDNA microarrays containing 41 421 elements, corresponding to 27 290 different UniGene cluster IDs. The hybridisations were performed as described previously, using 2 *μ*g of sample mRNAs and 2 *μ*g of reference mRNAs from 11 human cancer cell lines (Schaner *et al*, 2003). The arrays were scanned using the GenePix 4000A microarray scanner (Axon Instruments, Union City, CA, USA).

### Immunofluorescence staining

Cells were grown on LAB-TEK II chamber slides (Thermo Fisher Scientific), fixed for 20 min in 4% (v/v) paraformaldehyde and washed several times with PBS. The cells were blocked with 2% (w/v) BSA in PBS for 1 h at room temperature, incubated in an anti-Vimentin antibody (1 : 200, Abcam, Cambridge, MA, USA) at 4 °C overnight, and recognised by a donkey anti-mouse IgG-Alexa 546 antibody (1 : 400, Thermo Fisher Scientific). The slides were dried overnight after applying ProLong Gold Antifade Mountant with DAPI (Thermo Fisher Scientific), and then visualised on a Nikon E800 fluorescence microscope (Melville, NY, USA).

### Immunoblotting

Immunoblotting was performed using the following antibodies: anti-Vimentin (clone RV202, Abcam); anti-E-cadherin (clone 67A4, Millipore); anti-class I, pan *α*- and *β*-tubulin (Sigma-Aldrich); anti-class II and III *β*-tubulin (Covance); class IV *β*-tubulin (Abcam); and specific antibodies for acetylated *α*-tubulin, p53, p21 and GAPDH (Cell Signaling Technology). Membranes were then incubated with species-appropriate horseradish peroxidase-conjugated secondary antibodies for 1 h at room temperature prior to detection using the Clarity Western ECL substrate (Bio-Rad Laboratories, Hercules, CA, USA).

### Migration assay

Wound-healing assays were performed in triplicate measurements using the CytoSelect 24-well Wound-Healing Assay (Cell Biolabs, Inc. San Diego, CA, USA) according to the manufacturer’s instructions. After the wound-healing inserts were removed to expose the ‘wound fields’, cells were washed with complete medium to remove dead cells and debris, followed by replacement with medium containing 2% (v/v) fetal bovine serum. Photomicrographs were taken at baseline and 24 h after the inserts were removed.

### Quantitative PCR analysis

RT–qPCR using SYBR Green dye was performed to detect mRNA expression as described previously (Schaner *et al*, 2003), and amplimer sequences are listed in the Supplementary Table S2. RT–qPCR for mature miRNA was performed using miR-200b and miR-200c specific assays with the miScript System (Qiagen, Valencia, CA, USA) according to the manufacturer’s instructions.

### Statistical analyses

#### Data normalisation and filtering

Primary data collection was performed using GenePix Pro 3.0 (Axon Instruments). Array elements with obvious blemishes were manually flagged, and the raw data were submitted to the Stanford Microarray Database (SMD, Stanford, CA, USA). Data were background corrected, normalised using global intensity normalisation and filtered using a flag and background filter (1.5 minimal signal-over-background ratios in either channel). Only genes with at least 80% good data (fewer than 20% of array elements with blemishes or lacking target cDNA) were included in further analyses (Schaner *et al*, 2003). Next, the triplicate gene expression profiles of parental cell lines were averaged and the data matrix further transformed by separate centering of each parental cell line and corresponding resistant variants. A total of 31 arrays were analysed, corresponding to four parental cell lines hybridised in triplicate, and the hybridisations of the 15 drug resistant variants and four valspodar selected parental controls. We then proceeded with significance analysis of microarray (SAM, http://genome-www5.stanford.edu/).

#### Gene ontology and functional network analysis

Analyses of gene ontology, canonical pathways, and functional networks were executed using tools from Ingenuity Pathways Analysis (IPA, Ingenuity Systems, Mountain View, CA, USA). See ‘Script for Bioinformatic Analyses’ in the Supplementary Information for complete details. For network analysis, IPA computed a score [*P*-score=−log_10_(*P*-value)] according to the fit of the set of supplied genes and a list of biological functions stored in the IPKB (IPA knowledge base). The score takes into account the number of genes in the network and the size of the network to approximate how relevant this network is to the original list of genes and allows the networks to be prioritised for further studies. A score >3 (*P*<0.001) indicates a >99.9% confidence that a gene network was not generated by chance alone (Bredel *et al*, 2005).

#### Clinical data analysis

To explore the clinical impact of the EMT phenotype, we analysed 11 well-known EMT marker genes in 226 chemo-naïve serous ovarian cancer samples that had advanced stage (II, III and IV) and grade (2 and 3) disease (Tothill *et al*, 2008), and applied average linkage clustering using the Cluster software (Eisen *et al*, 1998). The samples were segregated into two classes based on the non-supervised hierarchical clustering ‘dendrogram’, with up- and downregulated mesenchymal markers and transcription regulators (Supplementary Figure S6A). We then performed Kaplan–Meier Survival Analysis to explore the clinical implications of the two classes using GraphPad software (Supplementary Figure S6B and C).

#### *In vivo* bioluminescence imaging

Female nude mice (Charles River Laboratories, Hollister, CA, USA) were injected either subcutaneously (S.C.) or intraperitoneally (I.P.) with 5–10 × 10^6^ GLF-transduced GFP(+) OVCAR-3 parental or OVCAR-3/TP variant cells and imaged twice weekly (Moisan *et al*, 2014). At each time point, all mice were injected I.P. with 3 mg D-Luciferin Firefly per mouse (Biosynth International, Itasca, IL, USA), anesthetised for 10 min with isoflurane, then imaged using the IVIS Spectrum imaging system (Perkin Elmer, Waltham, MA, USA). Animal procedures were approved by Stanford University’s Administrative Panel on Laboratory Animal Care, which is accredited by the Association for the Assessment and Accreditation of Laboratory Animal Care (AAALAC International).

## Results

### Taxane- and PSC-selected variants are not resistant due to transporters

Paclitaxel- and docetaxel-selected variants, counter-selected with PSC, were designated with TP and TxTP respectively. *ABCB1* expression by RT–PCR was at parental levels in each of the 8 TP and TxTP variants, and the lack of P-gp expression was confirmed by C219 immunoblotting and UIC2 staining by flow cytometry (data not shown). In addition to *ABCB1*, we also profiled 31 non-P-gp transporter genes (listed in Supplementary Online Material) in the TP and TxTP variants from the four parental lines. Expression of the non-P-gp transporters was not upregulated in these variants.

The eight TP and TxTP resistant variants displayed a non-MDR-resistant phenotype with substantial levels of resistance to taxanes (Table 1) that was not reversed by valspodar, and no decrease in cellular taxane accumulation. Cytotoxicity dose–response curves for the 1A9/TP80 variant with and without PSC, as an example, are shown in Supplementary Figure S1. There was no consistent cross-resistance phenotype for DNA-targeted drugs among these variants, except for the two OVCAR-3 variants that showed substantial cross-resistance to doxorubicin, gemcitabine, and topotecan (Supplementary Table S1).

### Reduced tubulin polymer in taxane variants and altered tubulin dynamics

**Figure 1:**
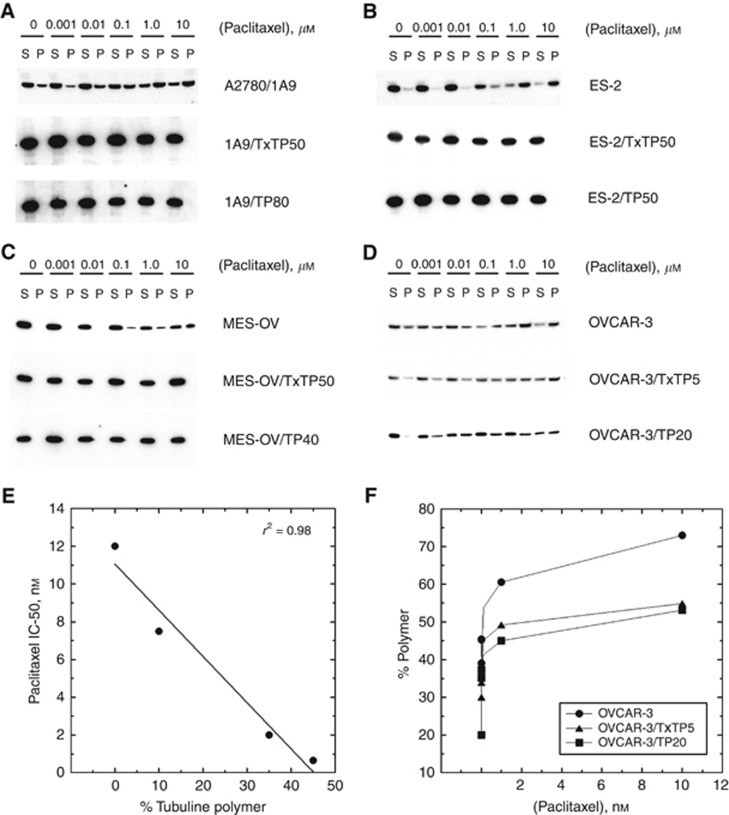
**Expression of tubulin polymer in response to treatment with paclitaxel in parental and taxane-resistant variants.**Soluble tubulin (S) and tubulin polymer (P) fractions from A2780/1A9 (**A**), ES-2 (**B**), MES-OV (**C**), and OVCAR-3 (**D** and **F**) cells were isolated by centrifugation (20 000 *g*) following a 5 min drug incubation in hypotonic buffer. Equal volumes of each fraction were resolved on gradient polyacrylamide gels and transferred to nitrocellulose. Immunoblotting with a pan *α*-tubulin antibody (clone DM1A, Sigma Aldrich) isolated the tubulin components. Following densitometry, the percentage of tubulin polymer present was calculated relative to total tubulin (soluble and polymerised). The relationship between the percentage of tubulin polymer present in untreated parental cells and paclitaxel activity as measured by the IC_50_ in our SRB cytotoxicity assays was determined (**E**).

The majority of the tubulin was present in the soluble form under our assay conditions, and we observed a dose-dependent increase in tubulin polymer content with acute paclitaxel exposure in the parental cell lines (1 to 10 nmol l^−1^, Figure 1A–D). Under identical experimental conditions, there was a reduction in tubulin polymer present in the all eight taxane-resistant variants compared to the parental controls, and in three of these variants (1A9/A2780, ES-2 and MES-OV), treatment with paclitaxel did not result in tubulin polymer expression up to the highest paclitaxel concentration tested (Figure 1A–C).

There was an inverse correlation (*r*^2^=0.98) between the percentage of tubulin polymer present in the four untreated parental cell lines and paclitaxel activity determined by IC_50_ in SRB assays, with the cell line having the most tubulin polymer being the most sensitive to paclitaxel (OVCAR-3), and the least sensitive having an undetectable amount of tubulin polymer (MES-OV, Figure 1E). In OVCAR-3 cells, we observed a reduction in tubulin polymer in the OVCAR-3/TxTP5 and OVCAR-3/TP20 variants, and the effects of paclitaxel were reduced compared to parental OVCAR-3 (Figure 1D and F).

### Reduced tubulin polymer is associated with reduced intracellular taxane content

**Figure 2:**
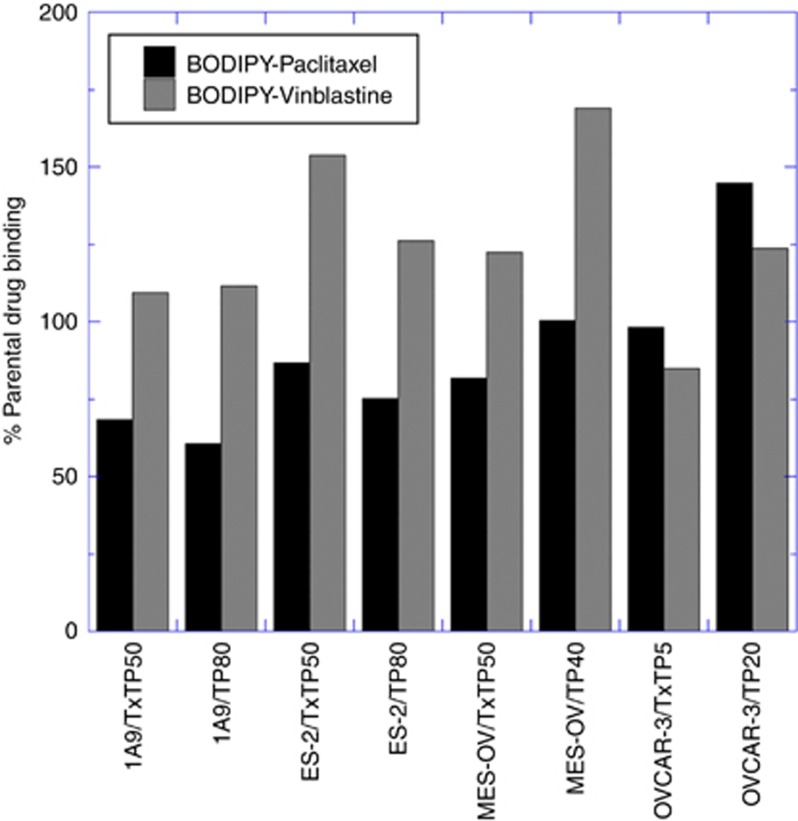
**Intracellular drug patterns in the taxane-resistant variants compared to parental controls.**Cells were allowed to accumulate either fluorescent BODIPY-paclitaxel or BODIPY-vinblastine for 1 h at 37 °C in 1x Hank’s balanced salt solution (HBSS) with 10 mM Hepes buffer without Phenol Red (Thermo Fisher Scientific). Following a 1 h drug-free efflux, cells were washed twice with 1x HBSS to remove any unbound drug. 50 000 events per condition were collected by flow cytometry and data expressed as a percentage of intracellular drug in parental cells.

Although the non-MDR taxane variants accumulated equivalent levels of taxanes relative to parental controls (data not shown), five out of eight variants had reduced intracellular BODIPY-taxane compared to parental controls (Figure 2). The association between resistance to paclitaxel as measured by the IC_50_ and drug content was inversely correlated (*r*^2^=0.84). Conversely, seven of eight variants that were hypersensitive to the effects of tubulin destabilisers (Table 1) demonstrated elevated BODIPY-vinblastine relative to parental controls (*r*^2^=0.72). One outlier is the OVCAR-3/TxTP5 variant that demonstrated a unique phenotype of resistance to both tubulin polymerising and destabilising agents.

### Elevated TUBB3 content in the majority of the variants

**Figure 3:**
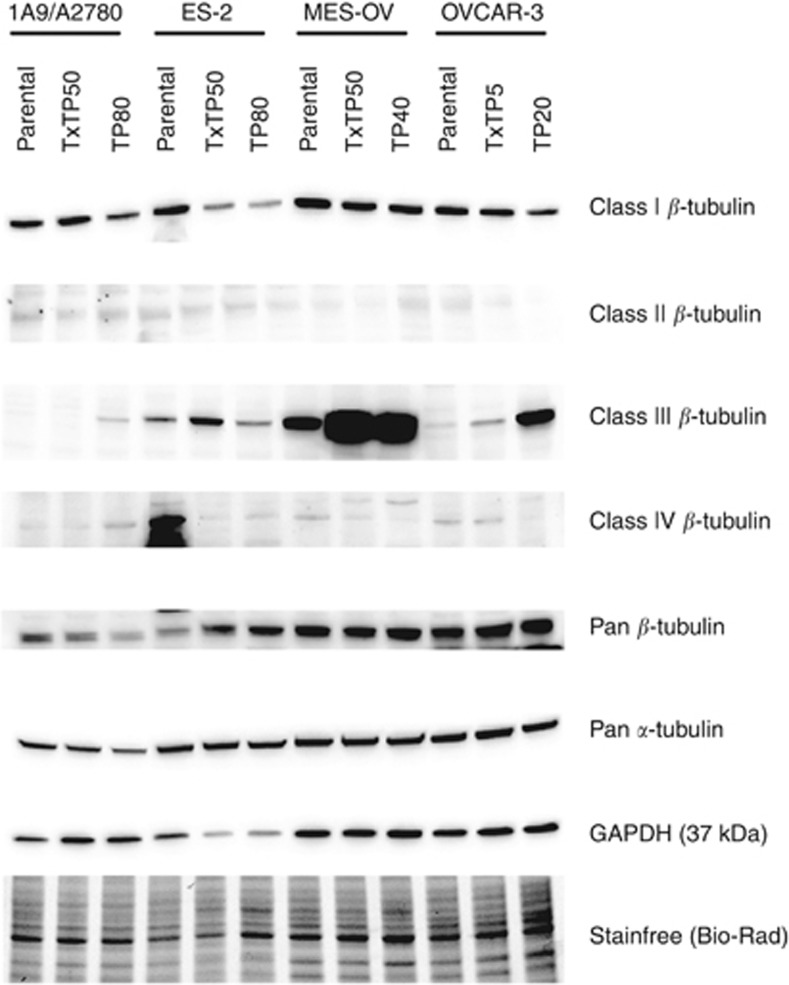
**Expression of class-specific *β*-tubulins by immunoblotting.**Total cell lysates were prepared in 1x RIPA buffer with freshly added protease inhibitors (cocktail from Bio-Rad). Proteins were separated by 4 to 20% gradient polyacrylamide gels, transferred to nitrocellulose, then blocked in 1x TBS containing 5% (w/v) nonfat milk and 1% (w/v) BSA for 1 h. Primary antibodies were incubated overnight at 4 °C and recognised by species-appropriate HRP-conjugated secondary antibodies. Bands were detected by Clarity Western ECL substrate on a ChemiDoc MP image analyzer (all Bio-Rad). Protein loading was normalised by both GAPDH expression and by stain-free visualisation following a 1 min UV-induced reaction to produce fluorescence in TGX gels (Bio-Rad) prior to membrane transfer.

Tubulin content was profiled by RT–qPCR and confirmed by immunoblotting with tubulin class-specific antibodies. We observed a reduction in the predominant *β*-tubulin isotype (class I, TUBB, Figure 3) in seven variants compared to parental controls and normalised to both GAPDH expression and to total protein using Stain-Free TGX gels (Bio-Rad Laboratories). Although we observed reduced TUBB content, no mutations in the isotype were detected by Sanger DNA sequencing in both forward and reverse directions, and by denaturing high-performance liquid chromatography (Sale *et al*, 2002), data not shown. The expression of *β*-tubulin classes II and IV was low to undetectable depending on the cell lines tested. Six variants had elevated class III (TUBB3) expression following drug selection, but overall pan *α*- and *β*-tubulin expression appeared stable compared to parental cell lines. Finally, when stabilised by taxanes, microtubules are acetylated (Gu *et al*, 2016), and we observed a reduction in total acetylated *α*-tubulin (Lys40) in all taxane variants (data not shown).

### Supervised gene expression analysis identifies EMT and cell cycle regulators associated with non-MDR taxane resistance

**Figure 4:**
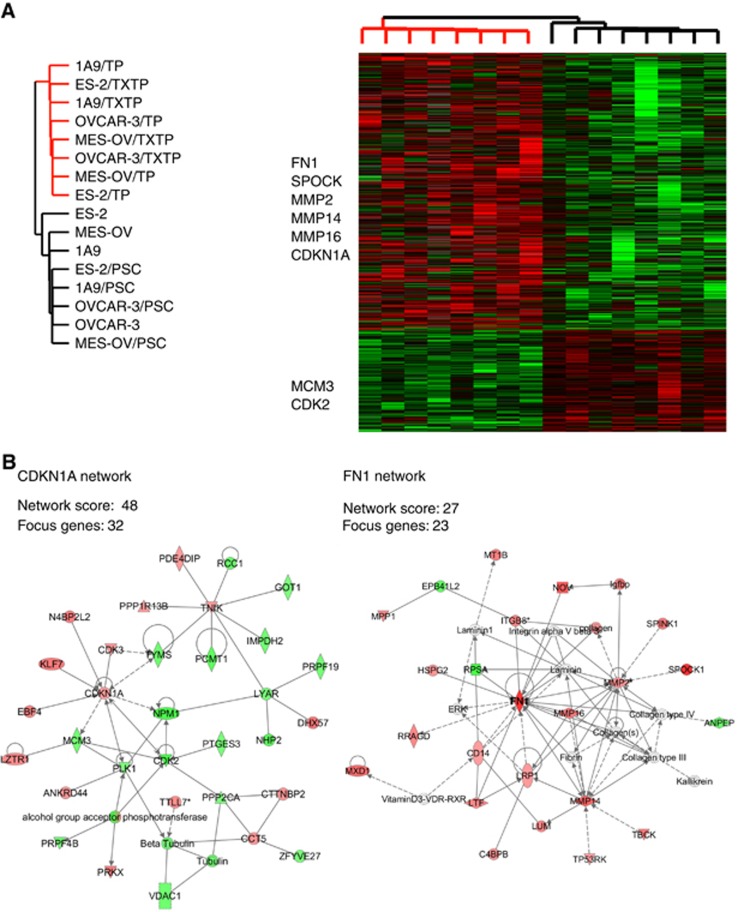
**Hierarchical clustering of supervised analysis by SAM differentiated taxane-resistant variants from parental cells.**28 225 clones were analysed by SAM, resulting in 1304 clones representing significantly differentially expressed genes between non-MDR and control samples (**A**). Differentially expressed genes are shown in Treeview. Each row represents a separate cDNA clone on the microarray, and each column represents a separate mRNA sample. Bright red represents the highest levels and bright green the lowest levels of expression. In each sample, the ratio of abundance of transcripts of each clone to its cell line adjusted mean abundance is depicted according to the colour scale shown at the bottom. The dendrogram at the top of the figure represents the hierarchical clustering of the samples based on similarity in their patterns of gene expression. Functional networks were identified using Ingenuity Pathways Analysis of genes identified by SAM. Depicted are network maps of molecular interactions and subcellular distribution of two networks, *FN1* and *CDKN1A*, that distinguish taxane-resistant from drug sensitive cells (**B**). Nodes represent genes, with their shapes representing the functional classes of the gene products, and edges indicate the biological relationship between the nodes, which include physical and functional interactions. Nodes are colour-coded according to their *d*-score from SAM (red, overexpressed in TP and TxTP variants; green, underexpressed in TP and TxTP variants).

We applied a two-group SAM analysis to identify differentially expressed genes between the non-MDR taxane variants and control samples (parental cell lines and PSC alone-selected cells), and we identified 1304 clones (951 clones upregulated, 353 downregulated, Figure 4A) that clearly separated the two groups. Among the upregulated clones, 9.6% were associated with the extracellular region or cell adhesion. Of these, *SPOCK1* and *FN1* were the top genes upregulated in non-MDR-resistant variants (*d*-scores 3.84 and 3.38, respectively, *q*-value 0%). *FN1* is an important mesenchymal element participating in epithelial to mesenchymal transition (EMT; Mikheeva *et al*, 2010), and *SPOCK1* is one of the EMT-Core-Signature genes identified by Taube *et al* (2010). Other upregulated extracellular matrix (ECM)-interacting genes included *NOV*, *MMP2*, *MMP16*, *CDH6*, *COL12A1*, *COL15A1*, *LTF*, *LUM*, *ITGB8* and *CASK*. SAM also identified genes involved in cell cycle regulation and cytoskeleton organisation, including upregulated *CDKN1A* (p21), *TP53INP* and *TNIK*, and downregulated *TERT*, *MCM3*, *CDK2*, *RCC1*, *CDC6*, *NPM1* and *CDC16*. Several cytokine related genes were overexpressed in non-MDR models, such as *IL1F5*, *IL15*, *IL16* and *TLR1*, suggesting the involvement of proinflammatory elements in non-MDR resistance. Cells grown in PSC alone did not develop an EMT phenotype, and their gene expression clustered with parental cells not exposed to drugs.

### Network analysis reveals two key networks: *FN1* and *CKN1A*

To explore how the transcripts identified by SAM are related, the 1304 clones from SAM were first mapped to non-redundant elements in the Ingenuity knowledge base, and networks of interacting genes and their products were dynamically computed on the basis of individually modelled known relationships. The most significant enrichment was found for the genes involved in the function of cellular growth and proliferation (195 genes), cellular development (149 genes), cell cycle (86), cell–cell signalling and interaction (15 genes), and cell death (168 genes), Supplementary Table S3. Network analysis identified two highly significant networks with significance scores of 48 and 27 (Supplementary Table S4), one network built around *FN1* and another around *CDKN1A* (Figure 4B).

### EMT phenotype is associated with non-MDR taxane resistance

**Figure 5:**
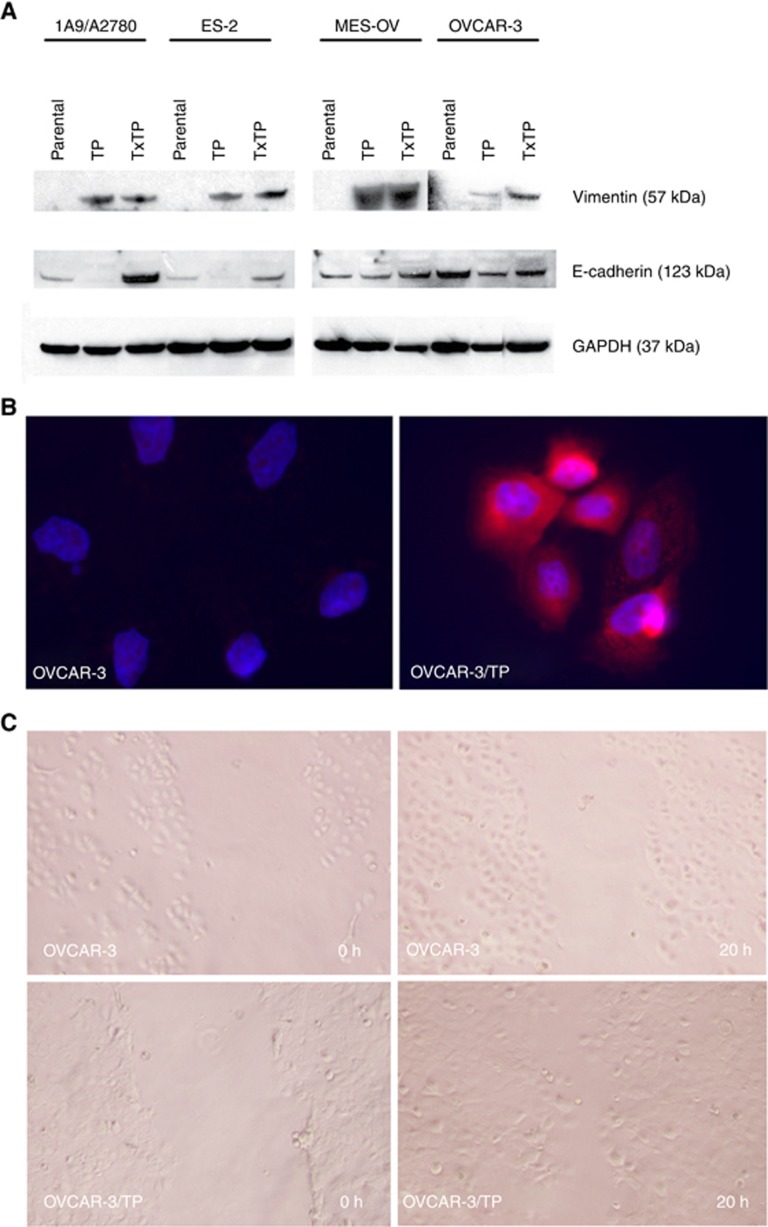
**Expression of EMT-related proteins in resistant variants, and cellular migration assays.**Protein expression of Vimentin and E-cadherin was analysed by immunoblotting in the eight taxane-resistant variants and 4 parental lines (**A**). GAPDH was used as internal control. Fluorescent immunocytochemistry of Vimentin in OVCAR-3 and OVCAR-3/TP cells (**B**). Cells were grown in LAB-TEK II chamber slides, and stained with antibody recognising Vimentin (red). DAPI (blue) is shown merged. A wound-healing assay was performed to determine the migration potentials between parental OVCAR-3 cells and OVCAR-3/TP20. Photos shown here were taken at 0, and 24 h after the wound gaps were made (**C**).

In addition to *FN1* and *MMP2* that were identified by genomic profiling, we confirmed the expression of several other EMT-associated genes using RT–qPCR. *MMP9* was markedly overexpressed in six variants at a range of 2- to 14-fold higher than controls (Supplementary Figure S2). *FN1* as a mesenchymal marker was identified by SAM analysis as a top upregulated gene in the non-MDR signature, and elevated expression was confirmed in the MES-OV and OVCAR-3 variants. *VIM* mRNA expression was increased in several resistant variants, and elevated Vimentin content was confirmed by immunoblotting in all eight resistant variants (Figure 5A) and by fluorescent immunocytochemistry in the OVCAR-3/TP cells (Figure 5B). Decreased E-cadherin protein was not concordant with the decreased Vimentin, with E-cadherin decreased in four and increased in four variants (Figure 5A). Cellular migration was markedly increased in the taxane variant of OVCAR-3 at 24 h compared to parental control as determined by a wound-healing assay (Figure 5C). Overall, six of the eight resistant variants manifested a strong EMT phenotype.

Of note, we did not find differences in mechanisms of resistance between the paclitaxel (TP) and docetaxel (TxTP) selected variants. EMT profiles were similar, and the TP and TxTP variants clustered together in hierarchical clustering of the microarrays.

### Altered miRNA 200 family members and apoptotic regulators

Cellular responses to taxanes are governed by several known factors. In particular, increasing evidence indicates an association between decreased expression of members of the miR-200 family, EMT, and increased *TUBB3* expression and taxane resistance (Park *et al*, 2008; Cochrane *et al*, 2009; Hu *et al*, 2009). We observed a marked decrease in both miR-200b and miR-200c in five of the resistant variants and a moderate decrease in another (Supplementary Figure S3). We examined the expression of apoptotic regulatory genes, and six of the eight resistant variants manifested increased *BCL-2* and *MCL-1* content by qPCR (Supplementary Figure S4). Both OVCAR-3/TP and TxTP variants have upregulated *BCL-XL*. The proapoptotic gene *BAX* was below control levels in two resistant variants.

### *TP53* status and non-MDR taxane resistance

We assessed the *TP53* status of these cells and their response to the DNA damaging drug doxorubicin (Dox), Supplementary Table S5 and Supplementary Figure S5. Treatment with Dox resulted in p53 accumulation in the 1A9/A2780 cell line and its taxane variants and downstream activation of p21, indicating a functional p53. ES-2, MES-OV, and OVCAR-3 cells and their variants had high levels of p53 with and without Dox. We could not detect p21 following DNA damage in ES-2, and p21 levels were low in MES-OV and OVCAR-3. Sequencing *TP53* revealed mutations in ES-2 (Exon 7: Ser-241 to Phe, TC^843^C→TTC) and MES-OV (Exon 8: Arg-282 to Trp, C^965^GG→CAG). In OVCAR-3 cells, we confirmed a single *TP53* mutation resulting in Arg-248 to Gln substitution (CG^864^G→CAG). Overall, there was no difference between parental cells and their taxane-resistant variants in this pathway.

### Proliferative potential in taxane variants

The cellular doubling times *in vitro* were prolonged two-fold in three of the four resistant variants OVCAR-3TP, MES-OVCAR-3/TP and ES-2/TP, compared to the parental and valspodar treated cells (Supplementary Figure S6). The TxTP resistant variants also exhibited slower growth (data not shown). However, the growth of the resistant TP variants as xenografts in athymic nude mice was faster than the parental xenografts (Supplementary Figure S7).

### Clinical implications of EMT genes in ovarian cancer samples

We examined the expression of 11 EMT genes in publicly available microarray data from ovarian cancer samples (Tothill *et al*, 2008). To facilitate the analysis, we organised the gene expression patterns and samples by non-supervised hierarchical clustering and visualised in Treeview (Supplementary Figure S8A; Eisen *et al*, 1998). Remarkably, we observed a predominantly biphasic pattern of expression for the EMT genes in 226 chemonaïve serous ovarian cancer samples comprising advanced stage and grade tumours. The biphasic profile allowed a natural separation of these tumours into two classes—upregulated and downregulated. The mesenchymal markers *VIM*, *MMP2*, *SPOCK1*, *FN1*, *SPARC* and *MMP9* and the transcription regulators, *ZEB1*, *ZEB2*, *SNAI2* and *TWIST1* displayed similar expression pattern: upregulated in 105 samples and downregulated in 121 samples; but C*DH1*, the hallmark of EMT, showed an opposite expression pattern in most of the samples compared to the mesenchymal markers and transcription regulators. To explore the clinical implications of the observed EMT signatures, we ran Kaplan–Meier survival analysis to compare the two classes of samples. Compared to the downregulated class, tumours in upregulated class were significantly more likely to progress to metastasis and death in a 5-year follow-up period (*P*=0.0008 and 0.036 respectively, Supplementary Figure S8B and C).

## Discussion

The selection of drug resistance by exposure to taxanes alone *in vitro* results in transporter mediated mechanisms with *ABCB1* gene activation (P-gp) in the majority of cases (Wang *et al*, 2006). The prevalence of P-gp selection likely results from the high stringency of the selection process and the high degree of resistance conferred by P-gp. Since *ABCB1* expression is of uncertain clinical significance in ovarian cancers, including those resistant to taxanes, in this study we developed non-MDR-resistant variants by co-exposure of a panel of ovarian cancer cell lines to a taxane plus the P-gp inhibitor PSC. This ‘counter selection’ by PSC provided the desired stable, non-MDR, taxane-resistant variants to further study mechanisms of resistance to taxanes. A summary table of the alterations in these taxane plus PSC selected variants is presented as Supplementary Table S6.

Remarkably, but not surprisingly, all eight of the resistant variants selected demonstrated alterations in baseline and taxane-stimulated tubulin polymerisation (Dumontet and Sikic, 1999). This reduction in tubulin polymer was the common feature of all of the variants, hence a ‘hallmark,’ and in six of the eight was associated with increased class III *β*-tubulin expression. This finding related to selection for resistance also emphasises the central role of microtubule dynamicity in the mechanism of action of taxane drugs (Horwitz, 1994; Blagosklonny and Fojo, 1999; Orr *et al*, 2003).

Class III *β*-tubulin (*TUBB3*) is an important gene in taxane resistance and it is also a direct target of miR-200c (Cochrane *et al*, 2009). Although *TUBB3* was not identified as a differentially expressed gene associated with non-MDR resistance by genomic profiling, we reasoned that the cDNA platform we used might not be sensitive enough to discern the highly homologous tubulin isoforms. Therefore, we performed immunoblotting with isoform-specific antibodies, and found elevated class III *β*-tubulin in six of eight resistant variants, indicating that *TUBB3* may contribute to taxane resistance in these cells (Figure 3A). The most abundant *β*-tubulin tubulin isoform, *TUBB* (class I), was downregulated in the *CDKN1A* network, and its downregulation in all the variants was confirmed by RT–qPCR (data not shown) and by immunoblotting (Figure 3).

TUBB3 acts by reducing microtubule assembly, thus resulting in lower drug binding of docetaxel to microtubules (Hari *et al*, 2003). In our study, an inverse correlation between the resistance to paclitaxel and intracellular taxane content was highly significant (Figure 1). Furthermore, the majority of these variants are hypersensitive to tubulin destabilising drugs, and we observed higher vinblastine content in these variants compared to parental cells. The OVCAR-3/TxTP5 variant differs from the others in that it is resistant to both microtubule polymerising and tubulin destabilising agents, and has increased intracellular paclitaxel and vinblastine compared to parental OVCAR-3 cells.

We used genomic analysis to explore other alterations in these variants. SAM identified 1304 transcripts that have distinct expression profiles between non-MDR taxane-resistant variants and parental cells. The signature includes genes involved in EMT, ECM remodelling, and cell cycle regulation. Several mesenchymal elements were confirmed by qPCR to be overexpressed in resistant cells, including *FN1*, *VIM*, *SPARC*, *MMP9*, and *MMP2.* The mesenchymal phenotype was found in six of the eight non-MDR variants selected by taxanes*. FN1* was identified as a top-scoring up-regulated gene in non-MDR variants. The Ingenuity network built around *FN1* mainly involved regulation of cellular movement and cell-to-cell signalling. Fibronectin is involved in cell adhesion and migration processes. Its expression is correlated significantly with tumour stage in ovarian carcinomas, and has an adverse influence on overall survival (Franke *et al*, 2003). Overexpression of *FN1* confers resistance to multiple chemotherapeutic agents *in vivo* and *in vitro* in a variety of cell lines and tissues (Damiano *et al*, 2001; Haslehurst *et al*, 2012). These results highlight a role of extracellular matrix (ECM) proteins, particularly *FN1*, in conferring drug resistance in human cancers. ECM provides a survival advantage for tumour cells and promotes resistance in lung and ovarian cancer cells (Sethi *et al*, 1999; Sherman-Baust *et al*, 2003).

*FN1* is upregulated in EMT, a cellular trans-differentiation program that enables epithelial cancer cells to acquire a mesenchymal phenotype associated with high-grade malignancy, i.e. the ability to invade and metastasise (Taube *et al*, 2010). During EMT, downregulation of E-cadherin by regulators such as *SNAIL1*, *SNAIL2*, *ZEB1* and *ZEB2* results in the loss of cell-to-cell adhesion. A few studies have demonstrated an association between underexpressed E-cadherin and poorer survival rate in ovarian cancer patients (Davidson *et al*, 2000; Faleiro-Rodrigues *et al*, 2004). Cancer cells that have undergone EMT show increased resistance to apoptosis and paclitaxel (Kajiyama *et al*, 2007). Enhanced metastatic ability and chemoresistance are frequently concurrent during the therapeutic course, and are the major causes of poor prognosis of ovarian cancer patients. In our taxane-resistant models, the highly upregulated mesenchymal genes *FN1* and *MMP2* were associated with non-MDR taxane resistance. *SNAIL2* and *ZEB2*, master transcription regulators for EMT, were overexpressed in several resistant variants (data not shown). Furthermore, markedly increased migration potential was observed in the resistant cells. The downregulation of E-cadherin was confirmed by qPCR in the resistant variants (data not shown).

Upregulated *MMP14* and *MMP16* were observed in the *FN1*-associated network. *MMP14* (*MT1-MMP*) mediates the activation of *MMP2* (Sato *et al*, 1994). Upregulation of *MMP14* and *MMP16* (*MT3-MMP*) is involved in EMT (Gilles *et al*, 1997; Pulyaeva *et al*, 1997), and associated with the aggressiveness of human ovarian, breast and gastric cancer (Pulyaeva *et al*, 1997; Lowy *et al*, 2006; Moss *et al*, 2009). K-cadherin (*CDH6*) was also identified by our SAM analysis to be upregulated in the resistant models. The overexpressed mesenchymal cadherins may have a dominant effect in cell–cell interactions, and enhance the motility of tumour cells even in the presence of E-cadherin (Voulgari and Pintzas, 2009). The mesenchymal cell–matrix adhesion system is principally based on the integrin family, and several integrins were shown to be associated with EMT (Voulgari and Pintzas, 2009).

The miR-200 family is a marker for epithelial cells and a regulator of EMT (Park *et al*, 2008; Cochrane *et al*, 2009). MiR-200 maintains ‘epithelial-ness’ by directly targeting and suppressing *ZEB1* and *ZEB2*. Downregulation of the miR-200 family contributes to tumour metastasis (Park *et al*, 2008), and *FN1* has been identified as a novel target of miR-200c (Howe *et al*, 2011). Re-expression of miRNAs of the miR-200 family reduced expression of its target genes, including *ZEB1*, *ZEB2*, *FN1* and class III *β*-tubulin (*TUBB3*), and inhibited ovarian cancer cell migration and invasion (Cochrane *et al*, 2009; Hu *et al*, 2009). MiR-200c was shown to mitigate invasiveness of ovarian cancer cells and restore sensitivity to taxanes (Cochrane *et al*, 2009; Brozovic *et al*, 2015). In our study, miR-200b and miR-200c were downregulated in six of the resistant variants, and inverse expression pattern was observed between the miR-200 family members and mesenchymal genes. We have previously reported that the EMT phenotype was partially reversible with miR-200c and miR-141 mimics, but that effects on sensitivity to paclitaxel were cell-type dependent (Brozovic *et al*, 2015). Silencing TUBB3 using a specific siRNA SmartPool resulted in ∼2x sensitisation to paclitaxel but did not have an effect on the expression of EMT markers (unpublished data).

The position of tumour cells in the cell cycle in response to drug treatment plays an important role in the sensitivity of tumour cells to chemotherapy, and taxanes are more effective against proliferating compared to quiescent cells (Shah and Schwartz, 2001). In the network shown in Figure 4B, upregulated *CDKN1A* in the resistant variants interacts with a group of downregulated cell cycle regulatory genes, such as *MCM3*, *NPM1*, *TYMS*, *CDK2*, *PLK1*, *PTGES3* and *IMPDH2. CDKN1A* encodes a small 165 amino-acid protein also known as p21, a negative regulator of the cell cycle which mediates G1 growth arrest by both p53-dependent and -independent mechanisms (Abbas and Dutta, 2009). The activation of p21 and an ability to undergo p21-induced cell cycle arrest may explain in part the intrinsic chemotherapy resistance of mature teratomas (Mayer *et al*, 2003). Indeed, we observed substantial decreases in cell growth rate in resistant variants, with greater decreases seen in OVCAR-3/TP, MES-OV/TP and ES-2/TP cells. This decreased growth rate likely further contributed to the taxane-resistance phenotype of these cells.

*BCL-2*, *BCL-XL*, *MCL-1* and *BAX* are determinants of apoptosis in response to chemotherapeutic agents, and are variably expressed in ovarian cancers (Jones *et al*, 1998; Chun and Lee, 2004). The anti-apoptotic gene *BCL-2* was overexpressed in six of the eight resistant variants. Marked upregulation was seen in MES-OV taxane-resistant cells, with the greatest increase in MES-OV/TxTP, which was 62-fold higher than drug sensitive controls. *MCL-1* was also upregulated in six resistant variants, while two variants had reduced proapoptotic *BAX* content. *BCL-XL* was upregulated in the OVCAR-3/TP and TxTP variants, notable because of their cross-resistance to the DNA damaging drugs doxorubicin, gemcitabine, and topotecan (Supplementary Table S1). Although these changes in apoptotic genes may contribute to the taxane resistance of these variants, most of the variants did not show a broad cross-resistance phenotype.

Ovarian cancers with upregulated EMT genes conferred worse clinical outcomes, and had significantly shorter relapse-free and overall survival. Interestingly, the high stromal response subtype tumours (C1 group) identified by Tothill were all grouped in the red-cluster (upregulated). C1 was shown to have the poorest survival outcome as a group (Tothill *et al*, 2008).

In conclusion, we have developed a set of eight novel non-MDR taxane-resistant models by co-selection of ovarian cancer cells with taxane drugs and a P-gp inhibitor. The phenotypes of these resistant variants are complex, and include decreased tubulin polymerisation, elevated expression of TUBB3, EMT, a decreased rate of proliferation, and alterations in apoptosis regulatory genes. These alterations are linked to some extent at the regulatory level, that is, via the miR-200 family, but may also reflect independent selection events during prolonged drug selections. The EMT phenotype of ovarian cancer cells along with *TUBB3*, tubulin polymer, and apoptotic genes may serve as biomarkers for responsiveness to taxanes, and modification of these markers should be considered as therapeutic targets in cancers.

## Figures and Tables

**Table 1:** Resistance phenotype to tubulin-targeted drugs in taxane-resistant variants

	**Relative resistance**^a^						
	**microtubule stabilisers**			**Microtubule depolymerising drugs**			
**Cell line**	**Docetaxel**	**Paclitaxel**	**Ixabepilone**	**Vinblastine**	**Vincristine**	**Navelbine**	**Colchicine**
A2780/1A9/TxTP50	10±1.8	30±3.5	1.2±0.23	0.41±0.68	0.58±0.20	0.31±0.055	0.36±0.078
A2780/1A9/TP80	15±0.9	30±3.3	0.82±0.15	0.63±0.12	0.53±0.095	0.39±0.082	0.66±0.12
ES-2/TxTP50	4.8±0.35	15±1.8	3.0±0.50	0.43±0.066	0.28±0.050	0.42±0.067	0.90±0.23
ES-2/TP80	31±2.7	30±2.3	12±2.1	0.65±0.18	0.77±0.009	0.92±0.28	1.8±0.24
MES-OV/TxTP50	15±3.3	20±1.8	12±1.5	0.52±0.10	0.29±0.048	0.33±0.52	0.72±0.11
MES-OV/TP40	3.3±0.29	7.1±1.4	5.5±1.0	0.11±0.035	0.20±0.051	0.11±0.028	0.58±0.091
OVCAR-3/TxTP5	5.2±0.21	3.3±0.63	2.0±0.30	2.1±0.50	2.4±0.39	1.6±0.29	1.4±0.33
OVCAR-3/TP20	3.2±0.30	2.9±0.45	1.1±0.24	0.54±0.11	0.57±0.10	0.87±0.22	1.4±0.16

a Relative resistance to the respective wild-type cell line under the same conditions calculated by comparing the IC_50_ of the variant/IC_50_ of the wild-type cell line. Ratios of IC_50_’s relative to parental cells were determined by the SRB colorimetric cell proliferation assay following 72 h drug incubations.
