# Supplementary material for: Decreased levels of baseline and drug-induced tubulin polymerisation are hallmarks of resistance to taxanes in ovarian cancer cells and are associated with epithelial-to-mesenchymal transition
Source: Br J Cancer. 2017 Apr 11;116(10):1318–28. doi: 10.1038/bjc.2017.102 (PMC5482726; doi:10.1038/bjc.2017.102)
Supplement: Supplementary Information [file bjc2017102x1.pdf]

Decreased Levels of Baseline and Drug-Induced Tubulin Polymerization are Hallmarks of Resistance to Taxanes in Ovarian Cancer Cells

George E. Duran, Yan C. Wang, François Moisan, E. Brian Francisco, and Branimir I. Sikic

Supplementary Online Material

Submitted for publication in the *British Journal of Cancer* on November 8, 2016

Revision submitted on January 26, 2017.

Supplementary list of non-Pgp transporters whose expression was profiled in TP and TxTP variants from the four parental lines.

The non-Pgp transporter genes included the following: *ABCA1, ABCA12, ABCA2, ABCA3, ABCA4, ABCA5, ABCA6, ABCA7, ABCB10, ABCB5, ABCB6, ABCB7, ABCB8, ABCB9, ABCC1, ABCC10, ABCC2, ABCC3, ABCC4, ABCC5, ABCC8. ABCD1, ABCD3, ABCD4, ABCE1, ABCF1, ABCF2, ABCF3, ABCG1, ABCG2,* and *ABCG5.*

Table S1. Resistance phenotype to DNA-targeted drugs in taxane resistant variants.

Cell Line	Relative Resistance[1] Carboplatin	Doxorubicin	Gemcitabine	Topotecan
A2780/1A9/TxTP50	1.5 ± 0.33	1.4 ± 0.36	1.2 ± 0.42	1.1 ± 0.27
A2780/1A9/TP80	0.81 ± 0.12	1.1 ± 0.45	0.57 ± 0.14	0.77 ± 0.14
ES-2/TxTP50	2.3 ± 0.28	1.8 ± 0.31	1.7 ± 0.30	1.0 ± 0.30
ES-2/TP80	2.0 ± 0.51	0.16 ± 0.033	1.9 ± 0.39	1.8 ± 0.25
MES-OV/TxTP50	2.0 ± 0.62	1.5 ± 0.24	1.7 ± 0.25	1.4 ± 0.39
MES-OV/TP40	0.48 ± 0.091	2.0 ± 0.45	0.23 ± 0.034	2.1 ± 0.35
OVCAR-3/TxTP5	1.5 ± 0.28	3.7 ± 0.82	8.4 ± 1.4	4.2 ± 0.79
OVCAR-3/TP20	1.5 ± 0.19	6.7 ± 1.45	16 ± 3.1	6.7 ± 0.70

[1] Relative resistance to the respective wild-type cell line under the same conditions calculated by comparing the IC_{50} of the variant/IC_{50} of the wild-type cell line. Ratios of IC_{50}'s relative to parental cells were determined by the SRB colorimetric cell proliferation assay following 72 h drug incubations.

Table S2. Primer sequence for RT-qPCR.

Genes	Forward primer	Reverse primer
BAX	GACGGCCTCCTCTCCTACTT	CTCAGCCCATCTTCTTCCAG
BCL2	TGGCTGATATTCTGCAACACT	GGTGGCCAACTGGAGACTTA
BCLXL	CGGGCTCTCTGCTGTACATA	CCAGCAGCTCCTCACACATA
CDH1	TGAAGGTGACAGAGCCTCTGGAT	TGGGTGAATTCGGGCTTGTT
FN1	GGTGACACTTATGAGCGTCCTAAA	AACATGTAACCACCAGTCTCATGTG
MCL1	GGGAAAAACATGCAGTCCTC	TCCTGGCACAGCTATCAAAA
MMP2	TGATCTTGACCAGAATACCATCGA	GGCTTGCGAGGGAAGAAGTT
MMP9	CCCTGGAGACCTGAGAACCA	CCCGAGTGTAACCATAGCGG
SNAI2	ATGAGGAATCTGGCTGCTGT	CAGGAGAAAATGCCTTTGGA
TUBB3	CGAAGCCAGCAGTGTCTAAA	GCCTGGAGCTGCAATAAGAC
VIM	CCTTGAACGCAAAGTGGAATC	GACATGCTGTTCCTGAATCTGAG
ZEB1	AAGAAAGTGTTACAGATGCAGCTG	CCCTGGTAACACTGTCTGGTC
ZEB2	GCGGCATATGGTGACACACAA	CATTTGAACTTGCGATTACCTGC

Table S3. Gene ontology analysis of 1,304 clones associated with taxane resistance from SAM analysis.

Molecular and Cellular Function	p value▲	Number of genes*
Cellular Growth and Proliferation	5.87E-09 - 1.32E-02	195
Cell Death	6.09E-06 - 1.40E-02	168
Cell Cycle	1.01E-05 - 1.40E-02	86
Cellular Development	2.49E-05 - 1.32E-02	149
Cell-to-Cell Signaling and Interaction	3.42E-05 - 1.12E-02	15

▲Range of significances of the associated genes for the high-level function

* Number of genes significantly associated with the corresponding high-level function.

Table S4. Functional gene networks that were altered in taxane resistant variants compared to parental cells.

Network	Number of Focus Genes	Network Score[#]
FN1	23	27
CDKN1A	32	48

[#] Reflects the negative logarithm of the p value that indicated the likelihood of the focus genes in a network being found together as a result of random chance (99% confidence level; scores ≥ 2 were considered significant).

Table S5. *TP53* mutation status and sequence in the four parental lines.

Cell Line	*TP53* Mutation Status	*TP53* Sequence	Amino Acid	Exon
A2780/1A9	Wild-type			
ES-2	Mutant	$TC^{843}C$ -> TTC	Ser-241 -> Phe	7
MES-OV	Mutant	$C^{965}GG$ -> TGG	Arg-282 -> Trp	8
OVCAR-3	Mutant	$CG^{864}G$ -> CAG	Arg-248 -> Gln	6

Table S6. Summary of alterations in taxane with PSC selected variants.

	1A9/ TP	1A9/ TxTP	ES-2/ TP	ES-2/ TxTP	MES-OV/ TP	MES-OV/ TxTP	OVCAR-3/ TP	OVCAR-3/ TxTP
Decreased paclitaxel-induced tubulin polymerization (Figure 1)	**Yes**	**Yes**	**Yes**	**Yes**	**Yes**	**Yes**	**Yes**	**Yes**
Collateral sensitivity to Vincas (Table 1)	**Yes**	**Yes**	**Yes**	**Yes**	**Yes**	**Yes**	**Yes**	*No*
Increased BODIPY-Vinblastine binding (Figure 2)	**Yes**	**Yes**	**Yes**	**Yes**	**Yes**	**Yes**	**Yes**	*No*
Increased TUBB3 expression (Figure 3)	**Yes**	*No*	*No*	**Yes**	**Yes**	**Yes**	**Yes**	**Yes**
Increased Vimentin expression (Figure 4)	**Yes**	**Yes**	**Yes**	**Yes**	**Yes**	**Yes**	**Yes**	**Yes**
Decreased miR200 family members (Web Supplement Figure 4)	**Yes**	*No*	**Yes**	**Yes**	**Yes**	**Yes**	**Yes**	*No*
Increased p21 (Figure 5)	**Yes**	**Yes**	**Yes**	**Yes**	**Yes**	**Yes**	*No*	*No*
Decreased BRCA1 (Figure 5)	*No*	*No*	**Yes**	**Yes**	**Yes**	*No*	**Yes**	*No*

Titles and legends to supplementary figures.

Figure S1. **Cytotoxicity curves for paclitaxel in A2780/1A9 parental and its paclitaxel/PSC co-selected variant using SRB assays.** Each paclitaxel concentration was tested in quadruplicate, with and without 2 μmol/L PSC. The IC_{50} values (nmol/L) were determined directly from semilogarithmic dose-response curves.

Figure S2. RT-qPCR results of EMT genes. The measurements for each gene in the bar graphs were first normalized to *GAPDH,* which was calculated against the standard curves generated with a pool of cDNA from all the selection variants, and then normalized to the drug sensitive control cells.

Figure S3. Expression of miR-200 family members. Total RNA was isolated from sub-confluent growing cells with the use of Nucleo Spin miRNA Kit (Macherey-Nagel, Bethlehem, PA), and 1 μg RNA was used for first-strand cDNA synthesis by using the miScript II RNA Kit (Qiagen, Valencia, CA) according to the manufacturer's protocol. Real-time PCR using SYBR Green dye was performed to detect mRNA expression (miScript SYBR Green PCR Kit, Qiagen). For detection of miR-200 family members, the miScript Primer Assay (Qiagen) was used according to manufacturer's instructions, with RNU6 gene (Qiagen) used as an internal loading control, and then normalized to the parental control cells.

Figure S4. Expression of apoptosis regulatory genes by RT-qPCR. Amplimer sequences can be found in Supplementary Figure S1. Data is normalized to the expression of *GAPDH* and then compared to expression in parental controls.

Figure S5. Functional status of p53 following DNA damage. Cells were exposed to doxorubicin (Dox, 1 μmol/L for 24 hr) and cells were collected to screen for p53 induction by

immunoblotting using a specific monoclonal (DO-1) which recognizes both wild-type and mutant p53 under denaturing conditions. The parental ES-2, MES-OV and OVCAR-3 cells, and each of their respective variants also presented with high levels of p53, and with minimal or no p21 activation observed following DNA damage. All of the paclitaxel-selected variants derived from this panel of ovarian cell lines contained inactive p53, and sequence analysis confirmed mutations in previously reported in OVCAR-3 and identified *TP53* mutations in the ES-2 and MES-OV parental cell lines established in our laboratory (See Table S4). Both the non-MDR docetaxel- and paclitaxel-selected variants of the 1A9/A2780 cell lines maintained a wild-type p53 status, with p53 accumulation and activated p21 following Dox exposure. All blots were stripped of all antibodies and re-probed for protein loading controls.

Figure S6. Cell growth rates were determined by measuring cellular doubling times. 1,000 cells per well were seeded in 96-well plates at time 0, and cell numbers were recorded at 24, 48, 72, 96, 120 and 144 hours (6 wells for each time point). Growth rate was calculated for each sample using the formula: $T_d = (t_2 - t_1) * \log 2 / \log (OD_2/OD_1)$. A. Cell doubling times; B. Growth curves; C. Calculated growth rates. Lighter and darker grays represent taxane resistant variants and sensitive controls, respectively.

Figure S7. *In vivo* growth of the OVCAR-3 parental cells compared to OVCAR-3/TP paclitaxel resistant variants. A and B, Tumor burdens of groups of 5 mice injected subcutaneously (S.C.) or intraperitoneally (I.P.), measured by imaging of the bioluminescence after injection of luciferin. C, Photographs of two mice at day 38 after injection of 5×10^6 cells S.C. D, Photographs of two mice at day 26 after injection of 10×10^6 cells I.P.

Figure S8. EMT gene expression by microarray profiling and survival based on these signatures. A. Hierarchical clustering profile of 11 EMT genes (19 clones) is shown in

Treeview. Each row represents a clone on the Affymetrix U133 plus 2 platform, and each column represents a separate mRNA sample. Bright red represents the highest levels and bright green the lowest levels of expression. In each sample, the ratio of abundance of transcripts of each clone to its mean abundance across all the 226 samples is depicted according to the color scale shown at the bottom. The dendrogram at the top of the figure represents the hierarchical clustering of the samples based on similarity in their patterns of gene expression. The “Upregulated” class is represented by the dendrograms colored in red; and the “Downregulated” class is in green. B. Relapse free survival according to EMT gene expression status in 226 serous ovarian carcinoma patients from the Tothill database, $p = 0.0002$). C. Overall survival according to EMT gene expression status in 226 serous ovarian carcinoma patients from the Tothill database, $p = 0.0125$).

Supplementary Figure S1.

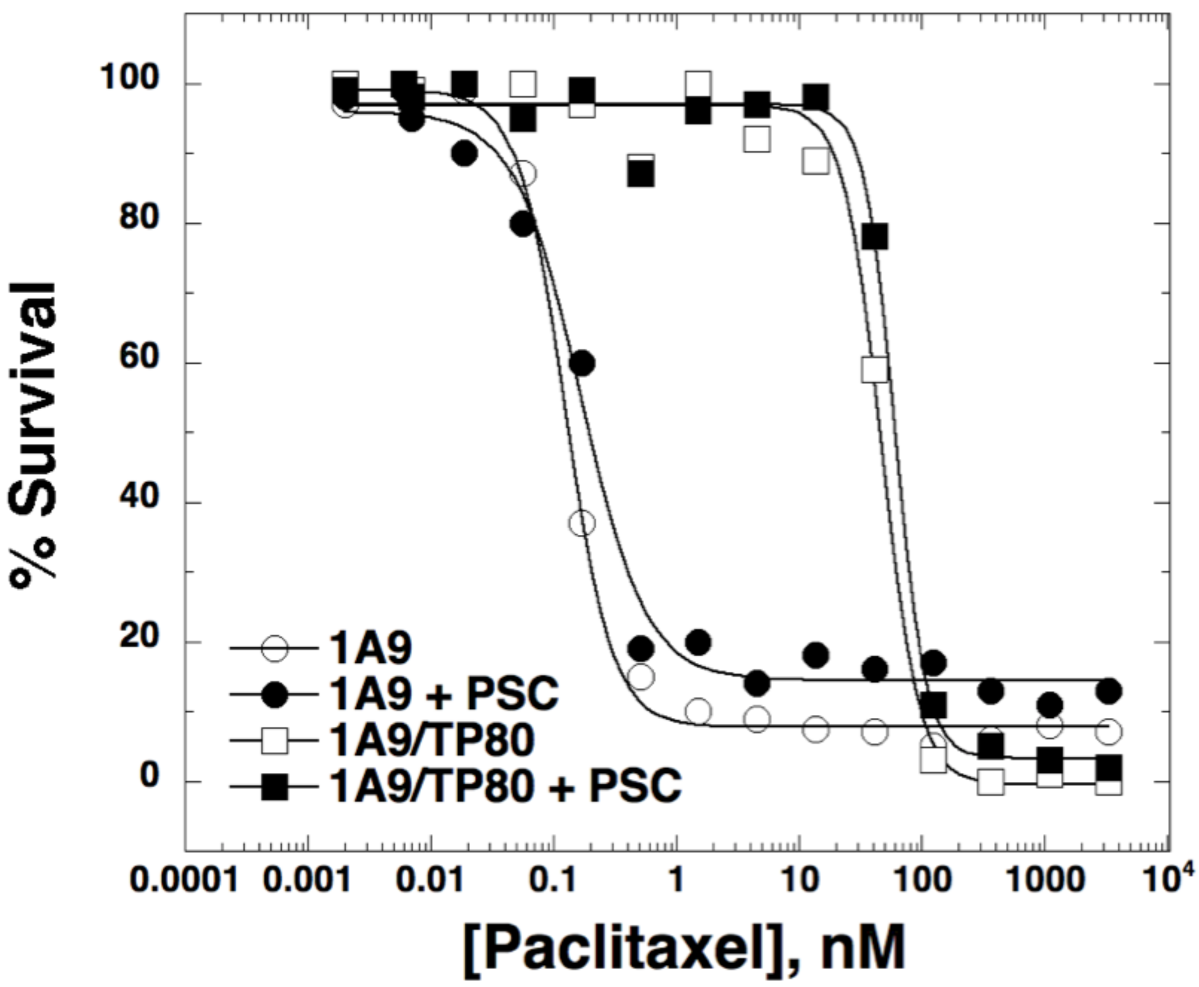

Supplementary Figure S2.

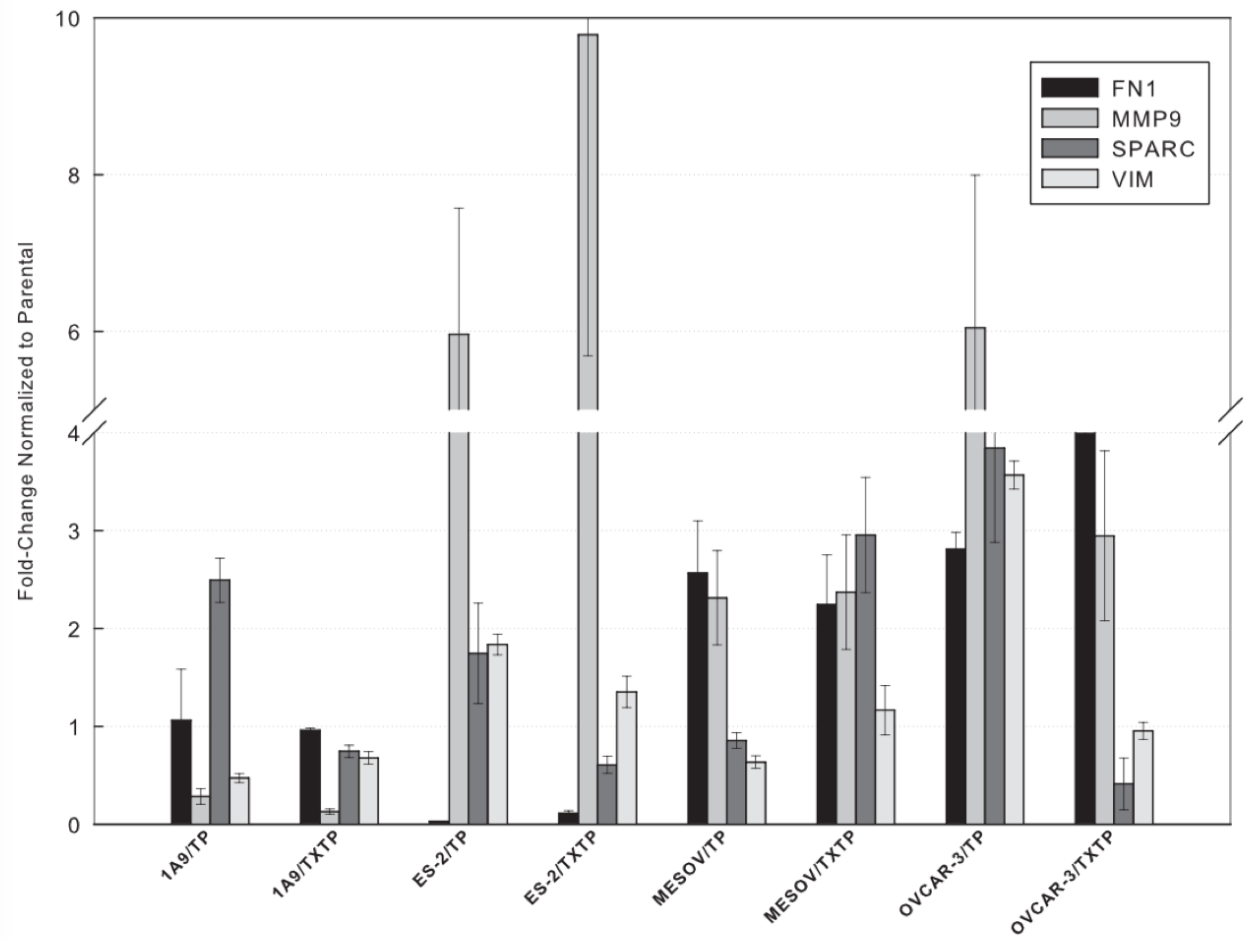

Supplementary Figure S3.

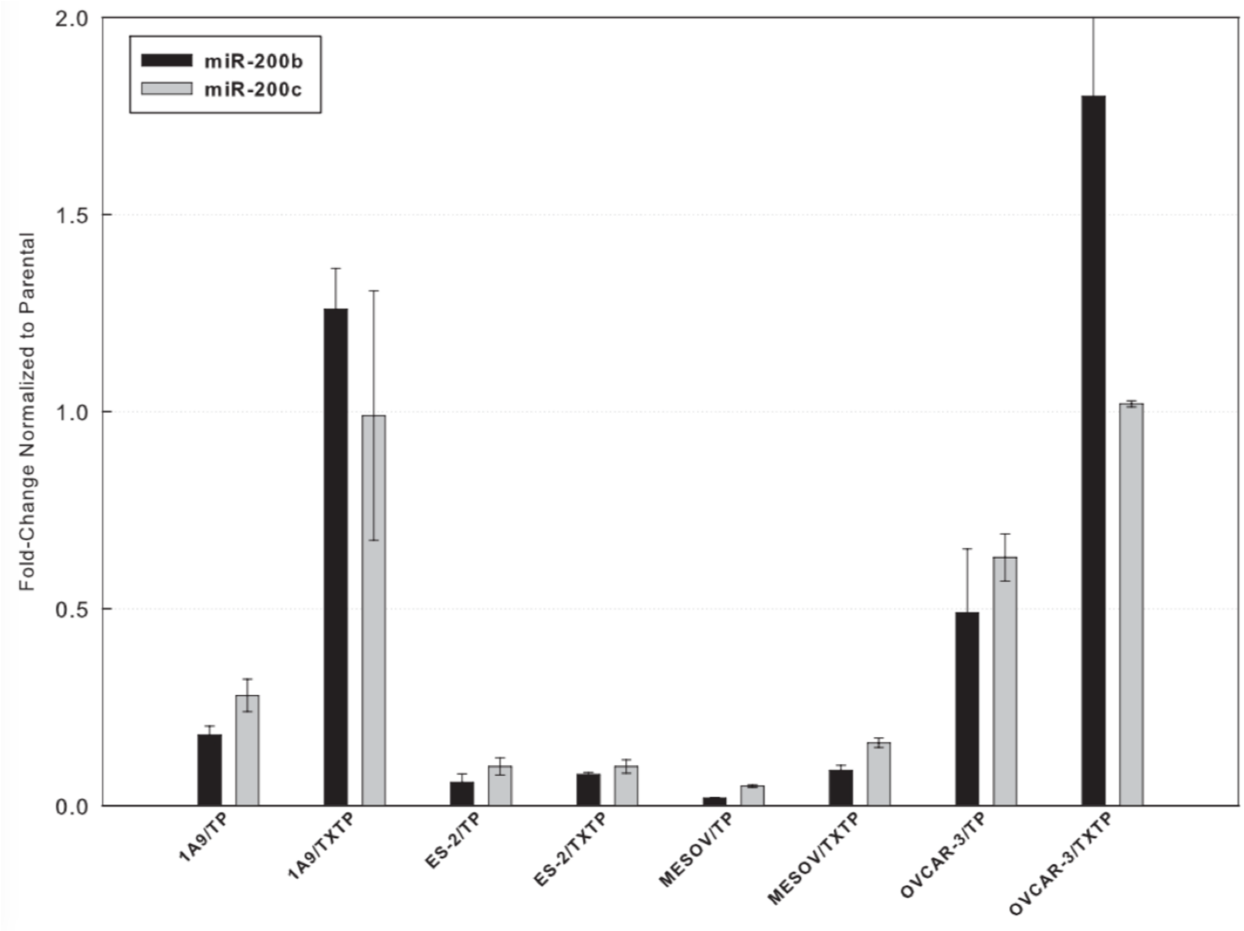

Supplementary Figure S4.

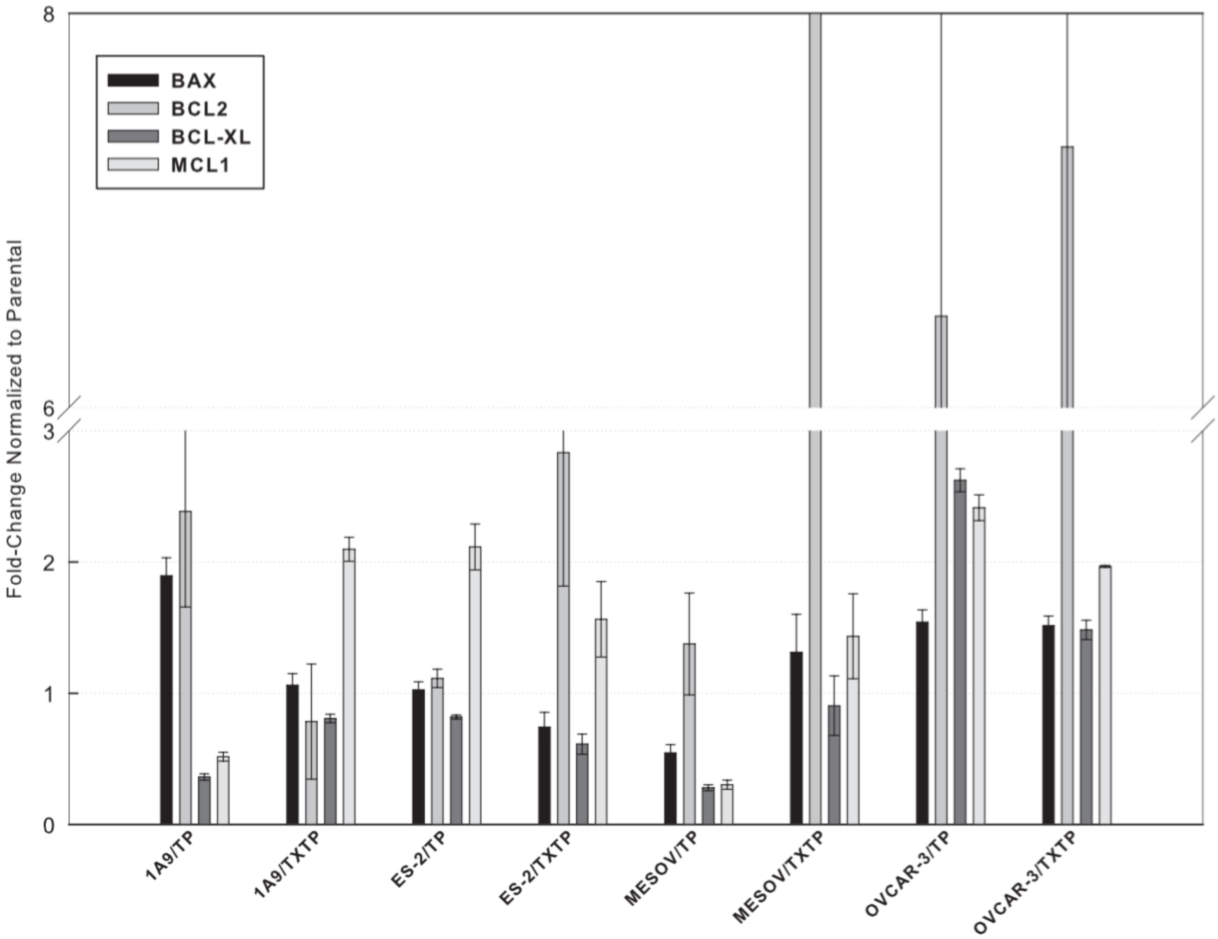

Supplementary Figure S5.

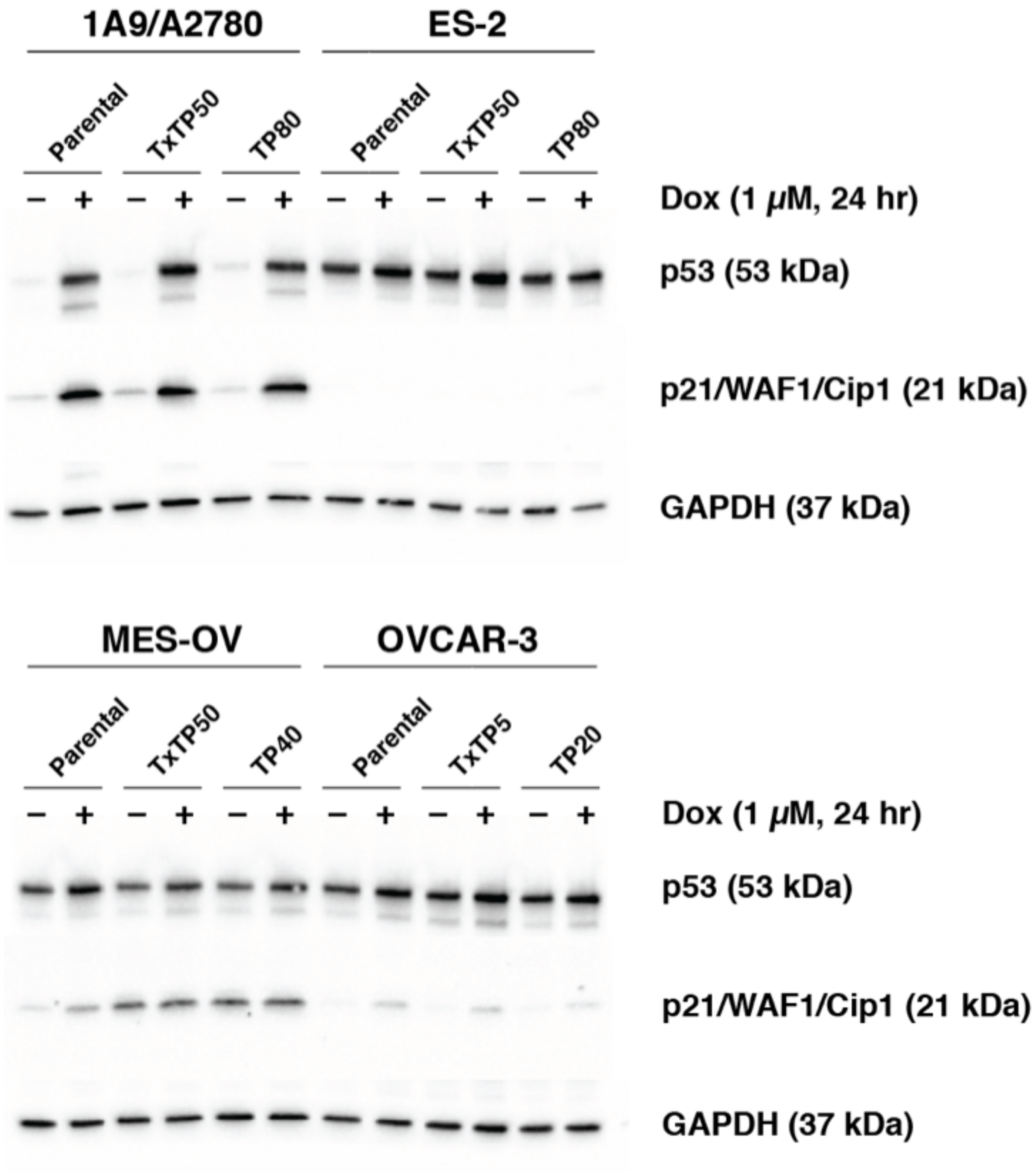

Supplementary Figure S6.

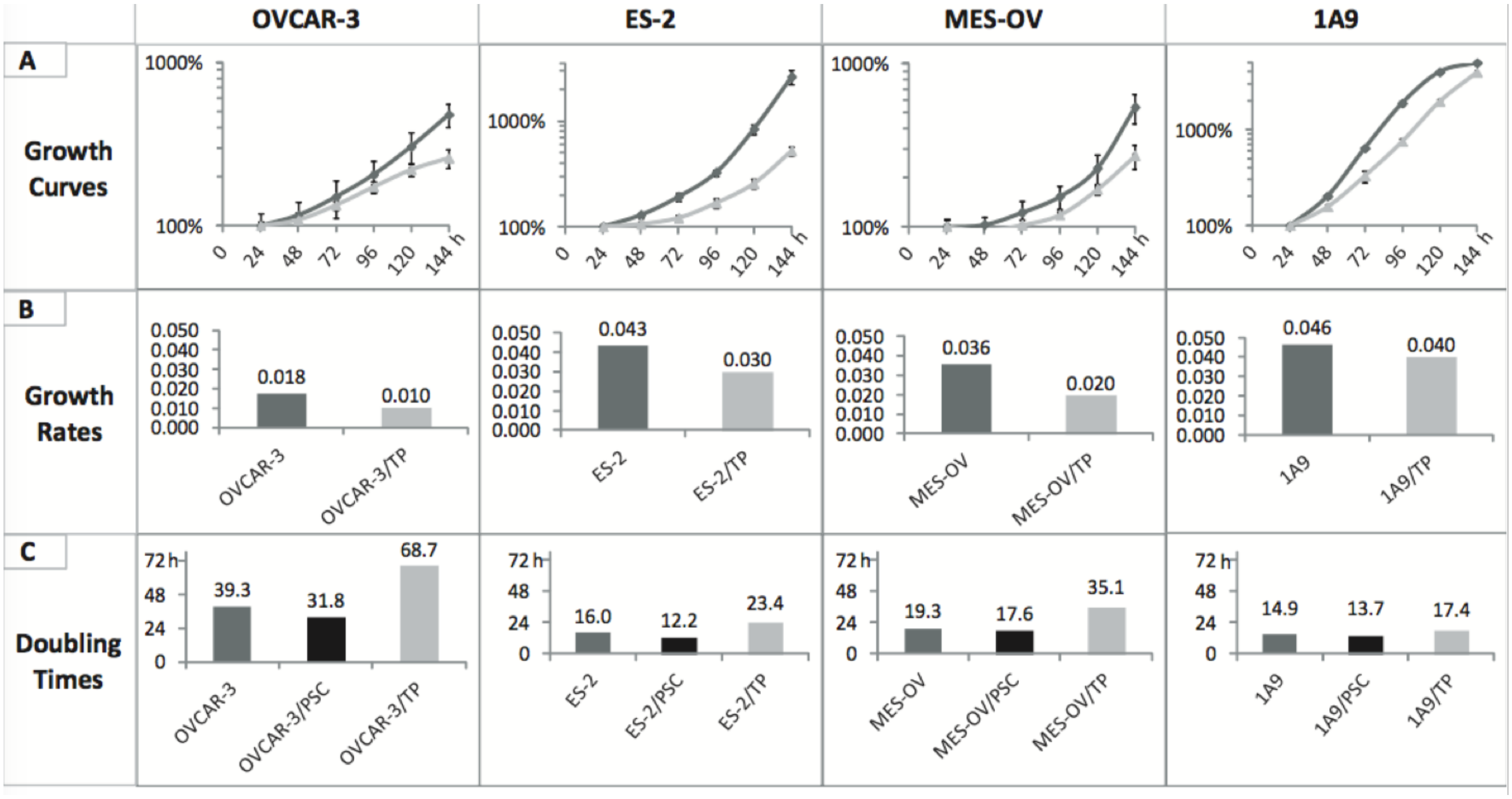

Supplementary Figure S7.

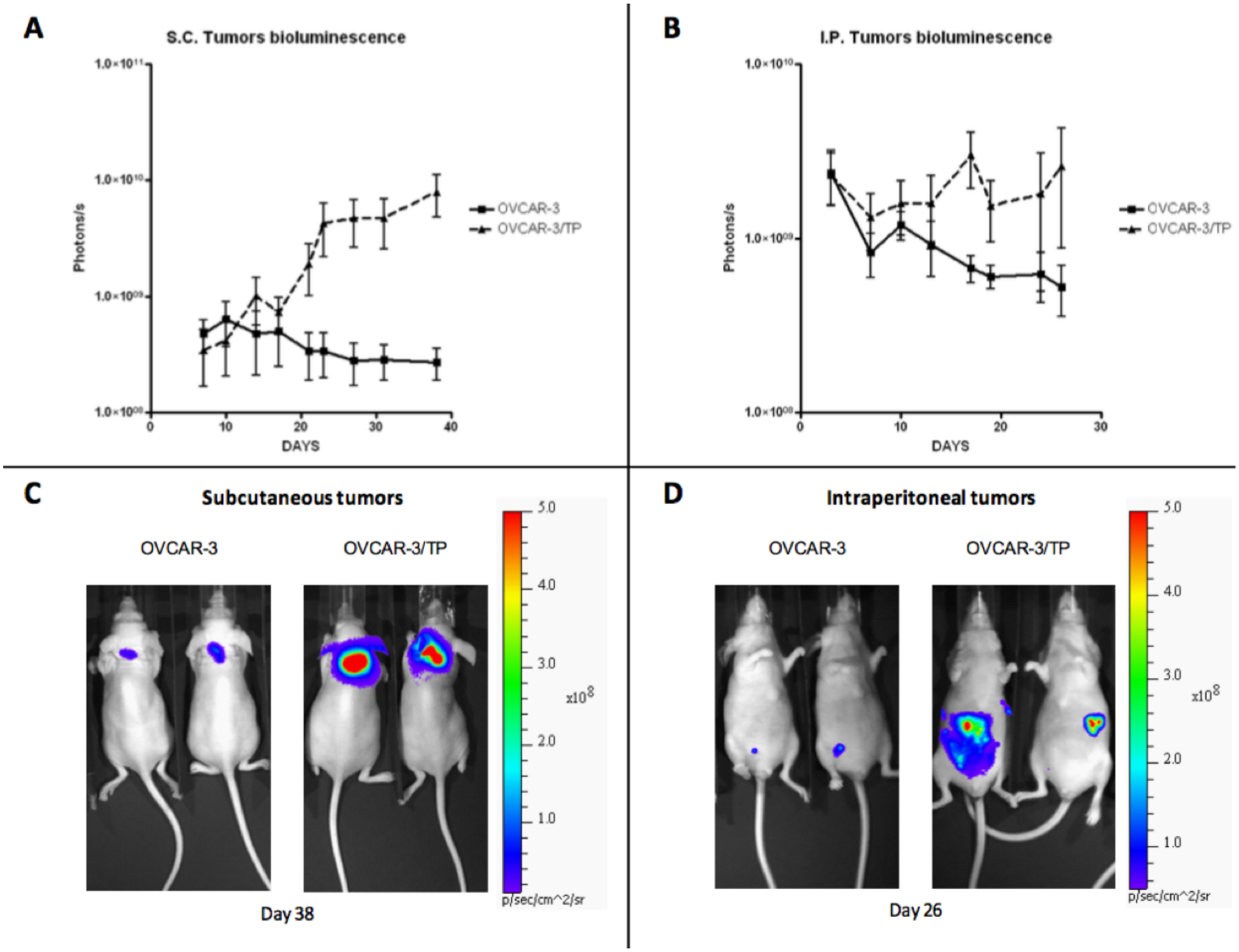

Supplementary Figure S8.

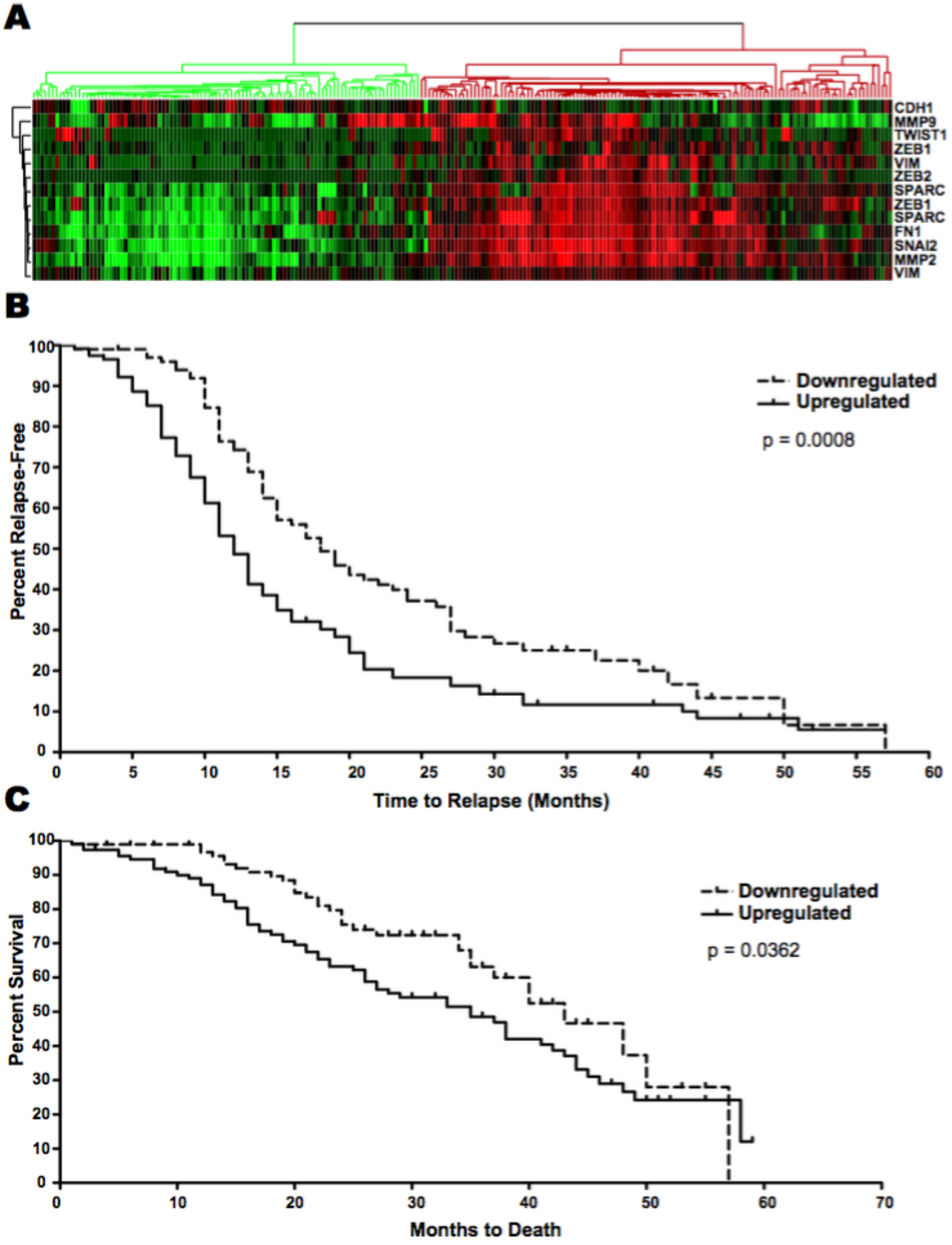

Script for Bioinformatic Analyses. mRNA from ovarian cancer cell lines was hybridized to Stanford human cDNA microarrays containing 41,421 elements, corresponding to 27,290 different UniGene cluster IDs. The arrays were scanned using the GenePix 4000A microarray scanner (Axon Instruments, Union City, CA). Primary expression data collection was performed using GenePix Pro3.0 (Axon Instruments). Array elements with obvious blemishes were manually flagged, and the raw data were submitted to the Stanford Microarray Database (SMD, Stanford, CA), background corrected, and normalized using global intensity normalization. Data were filtered using a flag and background filter (1.5 minimal signal-over-background ratios in either channel). Only genes with at least 80% good data were included in further analyses.

These criteria resulted in a list of 28,225 genes. Next, the triplicate gene expression profiles of parental cell lines were averaged and the data matrix further transformed by separate centering of each parental cell line and corresponding resistant variants. We then proceeded with SAM analysis. A total of 31 arrays were analyzed for this paper, corresponding to four parental cell lines hybridized in triplicate, and the hybridizations of the 15 drug resistant variants and four valspodar alone variants.

SAM was used to analyze differentially expressed genes between the non-MDR resistant *versus* the sensitive controls. The input file is the 28,225 clones after separate centering for each cell line. We applied two-group SAM (TP and TxTP versus parental and valspodar variants), centering arrays and other default options when running the program. The output of SAM analysis was a list of 1,304 clones at FDR 19.63%. At a higher stringency of less than 5% FDR, SAM generated a list of 49 genes. The SAM output containing accession numbers as identifiers for the 1,304 clones as well as d-scores were uploaded into the Ingenuity software for network interaction analysis (Ingenuity Systems, Mountain View, CA). Nodes represent genes in the

network, and are color-coded according their d-score from SAM (red, over-expressed in TPTxTP variants; green under-expressed in TPTxTP variants).

To explore the clinical impact of EMT phenotype, we analyzed 11 well-known EMT genes including mesenchymal markers and transcription regulators in a published ovarian cancer microarray dataset (GSE9891) (Tothill et al. *Clin Can Res,* 2008, 14; 5198). The data was generated on Affymetrix Human Genome U133 plus 2 platform. We downloaded RMA normalized data (in log format) for 285 specimens, and mapped the probes to the corresponding gene symbol using the NetAffy resource http://www.affymetrix.com/analysis/index.affx. We then extracted EMT genes from the 226 samples that had advanced stage (II, III, IV) and grade (2, 3) tumors. We further applied a thresholding filter which arbitrarily sets log expression values less than 5 to 5 to minimize the noise due to technically inadequate measurement. Next we mean-centered the expression data for each gene across all 226 arrays, and applied average linkage clustering using the Cluster software (Eisen et al., Proc Natl Acad Sci USA, 1998, 95; 14863). As shown in Supplementary Figure S8A, the samples were segregated into two classes based on the non-supervised hierarchical clustering "dendrogram"; and the class of samples with generally high levels of expression of mesenchymal markers and transcription regulators were classified as "Upregulated", while the other class was classified as "Downregulated". We then performed Kaplan Meier Survival Analysis to explore the clinical implications of the EMT genes in these two classes using GraphPad software following user's guide. The censored and missing data were automatically taken into consideration by the program. The upregulated tumors demonstrated significantly shorter relapse free and overall survival in 5-year follow-up period ($p = 0.0002$ and 0.0125, respectively) (Supplementary Figures S8B and S8C).
